# Bone Marrow CD8+ Cytotoxic T-lymphocyte Subset: A Prognostic Indicator in Acute Myeloid Leukemia

**DOI:** 10.7759/cureus.97217

**Published:** 2025-11-19

**Authors:** Randa A Osman, Abdallah M Almuslimani, Eman Z Kandeel, Marwa Hanafi, Mohammed A Samra, Azza M Kamel, Mahmoud A Ayoub

**Affiliations:** 1 Department of Clinical Pathology, National Cancer Institute, Cairo, EGY; 2 Department of Medical Oncology, National Cancer Institute, Cairo, EGY

**Keywords:** aml, antitumor immune response, bm t-lymphocytes, cd8+ ctls, flow cytometry

## Abstract

Acute myeloid leukemia (AML) is a heterogeneous disorder with variable clinical outcomes due to the complex molecular and cytogenetic basis of the disease. In light of recent developments in immunotherapy, bone marrow (BM) microenvironment immune composition, including baseline lymphocyte subset profiles, may be relevant for understanding host-tumor immunologic interactions. We conducted a study on 88 newly diagnosed AML patients to evaluate the baseline BM CD8⁺/CD3⁺ cytotoxic T-lymphocyte (CTL) subset by flow cytometry and its relation to standard prognostic factors, response to induction therapy, and disease outcome. Our results showed that a higher percentage of BM CD8⁺/CD3⁺ CTLs (≥10%) was associated with significantly better overall survival (P = 0.020) and progression-free survival (P = 0.020) compared with cases with <10%. Moreover, a lower BM CD8⁺/CD3⁺ CTL percentage was associated with adverse prognostic parameters, including higher total leukocyte count (r = -0.512, P < 0.001) and higher blast counts in both BM (r = -0.290, P = 0.006) and peripheral blood (r = -0.364, P < 0.001). These findings suggest an important role of this subset in the antitumor immune response. Large-scale studies are warranted to confirm these results and further clarify the role of BM CTLs as a prognostic indicator and predictor of survival in AML.

## Introduction

Acute myeloid leukemia (AML) is a clonal hematopoietic malignancy characterized by uncontrolled proliferation, impaired differentiation, and accumulation of immature myeloid precursors [1]. Clinical outcomes are highly variable due to the complex molecular and cytogenetic basis of the disease [2]. The primary goal of treatment is to control and, whenever possible, eradicate the disease. This is best achieved by establishing complete remission (CR) with initial therapy, followed by consolidation and/or maintenance strategies to deepen remission and maximize response duration [3]. The high relapse rate and poor outcomes in AML remain significant clinical challenges, highlighting the need for new diagnostic, predictive, prognostic, and therapeutic biomarkers [1].

AML pathogenesis is influenced not only by leukemic cells but also by abnormal bone marrow (BM) microenvironment profiles, which contribute to disease progression [4]. The immune system plays a key role in cancer prevention through immune surveillance, and observational studies have shown that immunocompromised individuals have higher rates of malignancies [5]. The physiological function of the immune system in tumors includes recognizing and eliminating clonally transformed cells before tumor development and destroying tumor cells after formation. It is now well established that the immune system mounts responses against many tumors, including AML, in vivo [6]. Different immune cell types contribute variably to antitumor activity. Natural killer cells and CD8⁺ cytotoxic T-lymphocytes (CTLs) mediate cytolytic responses against malignant cells, while B cells produce antitumor antibodies and exert additional inhibitory effects [7].

Adoptive immunotherapy, or adoptive cell therapy (ACT), involves isolating antigen-specific cells with direct anticancer activity, expanding and activating them ex vivo, and then administering them autologously. ACT is a highly personalized and promising cancer therapy that induces antitumor immune responses. In melanoma patients, ACT with naturally occurring tumor-reactive lymphocytes has mediated complete tumor regressions, potentially by targeting somatic mutations unique to each cancer. Furthermore, genetic engineering to induce expression of conventional T cell receptors or chimeric antigen receptors in T cells has expanded the therapeutic potential of ACT in cancer treatment [8].

The proportions of various immune cells in the BM differ across myeloid neoplasms, and their relative numbers at diagnosis may correlate with disease prognosis [9]. Therefore, understanding baseline lymphocyte subset composition in AML is crucial for the development of novel immunotherapeutic strategies. Although many studies have explored the population changes and prognostic significance of tumor-infiltrating and circulating T cells in solid tumors, research on BM T-cell subsets in AML remains limited [10].

In this study, we aimed to evaluate the baseline BM CD8⁺/CD3⁺ CTL subset in newly diagnosed AML patients and investigate its relationship with standard prognostic factors, response to induction therapy, and disease outcomes using multiparameter flow cytometry on fresh BM samples collected at diagnosis.

## Materials and methods

Patients

This study included 88 newly diagnosed AML patients who presented to the pediatric and medical oncology departments of the National Cancer Institute (NCI), Cairo University, between March 2021 and October 2021; 43 were male and 45 were female. All newly diagnosed AML patients during this period, including children and adults of both sexes, were eligible for inclusion. Exclusion criteria were the presence of other malignancies or previous chemotherapy or radiotherapy.

The study was conducted in accordance with the Declaration of Helsinki for the protection of human subjects. Written informed consent was obtained from all participants or their guardians. The study protocol was approved by the Institutional Review Board of the NCI, Cairo University (approval 201920069.3).

Methods

Patients were diagnosed using standard clinical, laboratory, and radiological methods. Hematological evaluation included CBCs, BM aspirates with French-American-British (FAB) classification, immunophenotyping, cytogenetics, and/or molecular genetic analysis. An acute leukemia immunophenotyping panel was used for diagnosis and AML subclassification.

Flow cytometry was performed using a 10-color BD FACS Canto (BD Biosciences, San Jose, CA, USA). BD Cytometer Setup and Tracking beads were used to monitor instrument performance, ensuring accurate and reliable data collection by tracking laser stability, signal detection, and resolution, and by optimizing detector voltages for consistent results. Standard acquisition protocols were followed for each experiment, with approximately 100,000 events acquired per sample. Data were analyzed using FACSDiva 8.0.2 software (BD Biosciences).

**Figure 1:**
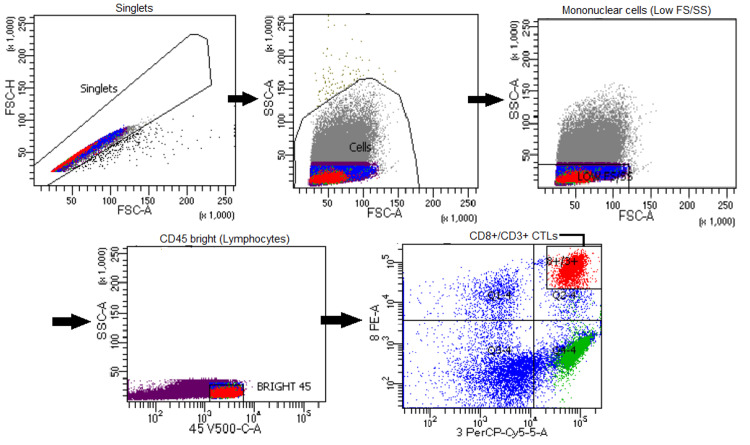
Flow cytometric gating strategy BM lymphocytes were gated based on bright CD45 expression. The gated lymphocyte population was then analyzed for the percentage of CD8⁺/CD3⁺ CTLs. BM, bone marrow; CTL, cytotoxic T-lymphocyte

Gating was performed on the residual BM lymphocyte population based on forward and side scatter properties and bright CD45 expression. The gated lymphocytes were then analyzed for the percentage of CD8⁺/CD3⁺ CTLs using the following monoclonal antibodies: PE-conjugated anti-CD8, PC5-conjugated anti-CD3, and Krome Orange (KrO)-conjugated anti-CD45 (Beckman Coulter Life Sciences, Indianapolis, IN, USA) (Figure 1). Patients were divided into high and low groups based on the median percentage of BM CD8⁺/CD3⁺ CTLs.

Response to therapy was assessed by the achievement of CR at the end of induction therapy. Survival outcomes were evaluated in terms of overall survival (OS) and progression-free survival (PFS). OS was defined as the time from diagnosis to death or last follow-up, while PFS was defined as the time from treatment initiation to relapse, death, or last follow-up.

Statistical analysis

Statistical analyses were performed using IBM SPSS Statistics for Windows, Version 22.0 (Released 2013; IBM Corp., Armonk, NY, USA). Numerical data were presented as mean ± SD or median and range, as appropriate. Qualitative data were expressed as frequency and percentage. The chi-square test was used to examine associations between qualitative variables. Correlations between numerical variables were assessed using Spearman’s rho correlation. Survival analysis was performed using the Kaplan-Meier method, and comparisons between survival curves were conducted using the log-rank test. All statistical tests were two-tailed, and a p-value < 0.05 was considered statistically significant. Patients were divided into high and low BM CD8⁺/CD3⁺ CTL groups based on the median percentage.

## Results

Clinical and hematological findings

Of the 88 patients, 27 were in the pediatric age group (≤18 years), and 61 were adults (>18 years). Pediatric patients had an age range of 0.6 to 18.0 years, with a median of 11.0 years and a mean of 10.1 ± 6.5 years. Adult patients ranged in age from 19.0 to 80.0 years, with a median of 47.0 years and a mean of 47.0 ± 16.7 years. Lymphadenopathy was observed in 35 of 88 patients (39.8%), hepatomegaly in 17 of 88 patients (19.3%), and splenomegaly in 17 of 88 patients (19.3%).

Initial hematological findings, including CBCs, peripheral blood (PB), BM blast percentages, BM lymphocyte percentages, and CD8⁺ T-lymphocyte percentages, are summarized in Table 1.

**Table 1:** Initial hematological parameters in 88 patients with AML AML, acute myeloid leukemia; BM, bone marrow; Hb, hemoglobin; PB, peripheral blood; PLT, platelets; TLC, total leukocytic count

Parameter	Mean ± SD	Median (range)
Hb (g/dl)	7.8 ± 1.7	7.6 (5.0-13.1)
TLC (x 10^9^/L)	60.0 ± 81.7	29.9 (1.3-419.4)
PLT (× 10^9^/L)	57.0 ± 55.0	41.0 (5.0-290.0)
PB blasts (%)	53.0 ± 27.0	53.0 (5.0-96.0)
BM blasts (%)	67.0 ±20.0	73.0 (20.0-98.0)
BM lymphocytes (%)	7.0 ± 6.0	5.0 (0-30.0)
CD8⁺/CD3⁺ (%)	12.6 ± 9.9	10.1 (0.6-43.9)

Most patients had a hypercellular BM (67/88, 76.1%), 19/88 patients (21.6%) had a normocellular BM, and 2/88 patients (2.3%) had a hypocellular BM. According to the FAB classification, M4 was the most frequent subtype (32/88, 36.4%), followed by M2 (29/88, 32.9%), M1 (20/88, 22.7%), M5 (5/88, 5.7%), and M0, which was the least frequent (2/88, 2.3%).

Molecular and cytogenetic findings

Most patients were in the intermediate cytogenetic risk group (22/42, 52.4%), while 15/42 (35.7%) were in the low-risk group, and 5/42 (11.9%) were in the high-risk group. Among molecular findings, eight of 54 patients (14.8%) were positive for t(8;22), 4/54 (7.4%) were positive for Inv(16), and 16 of 70 patients (22.9%) were positive for FLT3-ITD.

Associations of BM CD8⁺/CD3⁺ CTLs percentage with clinical and hematological parameters

A low percentage of BM CD8⁺/CD3⁺ CTLs was significantly associated with total leukocyte count (TLC) ≥ 100 × 10⁹/L (p = 0.011), hypercellular BM (p = 0.024), and M4/M5 FAB subtypes (p = 0.011). No significant associations were observed between initial BM CD8⁺/CD3⁺ CTL percentage and age (p = 0.632), gender (p = 0.522), lymphadenopathy (p = 0.828), hepatomegaly (p = 0.418), or splenomegaly (p = 0.177). Similarly, there was no association with hemoglobin concentration (p = 0.927), although cases with low BM CD8⁺/CD3⁺ CTL percentage showed a borderline association with platelet count ≥ 50 × 10⁹/L (p = 0.050).

BM CD8⁺/CD3⁺ CTL percentage was not significantly associated with molecular genetic abnormalities or cytogenetic risk groups. Furthermore, no significant difference was observed between responders and non-responders at the end of induction therapy (p = 0.734) (Table 2).

**Table 2:** Associations of BM CD8⁺/CD3⁺ CTL percentage with clinical and hematological parameters ^*^ Number of cases with available data for the parameter ^**^ Patients were divided into high and low BM CD8⁺/CD3⁺ CTL groups based on the median percentage. BM, bone marrow; CR, complete remission; CTL, cytotoxic T-lymphocyte; FAB, French-American-British classification; Hb, hemoglobin; PLT, platelets; TLC, total leukocyte count

Parameter		CD8⁺ T-lymphocyte groups^**^, N (%)		Statistical analysis	
		Low	High	Chi-square (χ²)	P value
Age groups	Pediatric	13 (54.2)	11 (45.8)	0.229	0.632
	Adults	31 (48.4)	33 (51.6)		
Gender	Male	20 (46.5)	23 (53.5)	0.409	0.522
	Female	24 (53.3)	21 (46.7)		
Lymphadenopathy	Absent	26 (49.1)	27 (50.9)	0.047	0.828
	Present	18 (51.4)	17 (48.6)		
Hepatomegaly	Absent	37 (52.1)	34 (47.9)	0.656	0.418
	Present	7 (41.2)	10 (58.8)		
Splenomegaly	Absent	33 (46.5)	38 (53.5)	1.823	0.177
	Present	11 (64.7)	6 (35.3)		
PLT (× 10^9^/L)	<50	22 (41.5)	31 (58.5)	3.843	0.05
	≥50	22 (62.9)	13 (37.1)		
TLC (× 10^9^/L)	<100	32 (43.8)	41 (56.2)	6.51	0.011
	≥100	12 (80.0)	3 (20.0)		
Hb (g/dl)	<8	26 (51.0)	25 (49.0)	0.047	0.829
	≥8	18 (48.6)	19 (51.4)		
BM cellularity	Hypo/normocellular	6 (28.6)	15 (71.4)	5.066	0.024
	Hypercellular	38 (56.7)	29 (43.3)		
FAB subtypes	M0-M1	7 (31.8)	15 (68.2)	6.489	0.011
	M2	13 (44.8)	16 (55.2)		
	M4-M5	24 (64.9)	13 (35.1)		
t (8;21) (N = 54)^*^	Negative	26 (56.5)	20 (43.5)	0.992	0.319
	Positive	3 (37.5)	5 (62.5)		
Inv(16) (N = 54)^*^	Negative	26 (52.0)	24 (48.0)	0.788	0.375
	Positive	3 (75.0)	1 (25.0)		
FLT3-ITD (N = 70)^*^	Negative	22 (40.7)	32 (59.3)	2.355	0.125
	Positive	10 (62.5)	6 (37.5)		
Cytogenetics (N = 42)^*^	High/intermediate risk	10 (37.0)	17 (63.0)	2.053	0.152
	Low risk	9 (60.0)	6 (40.0)		
CR at the end of induction (N = 78)^*^	Nonresponders	27 (50.0)	27 (50.0)	0.115	0.734
	Responders	13 (54.2)	11 (45.8)		

**Figure 2:**
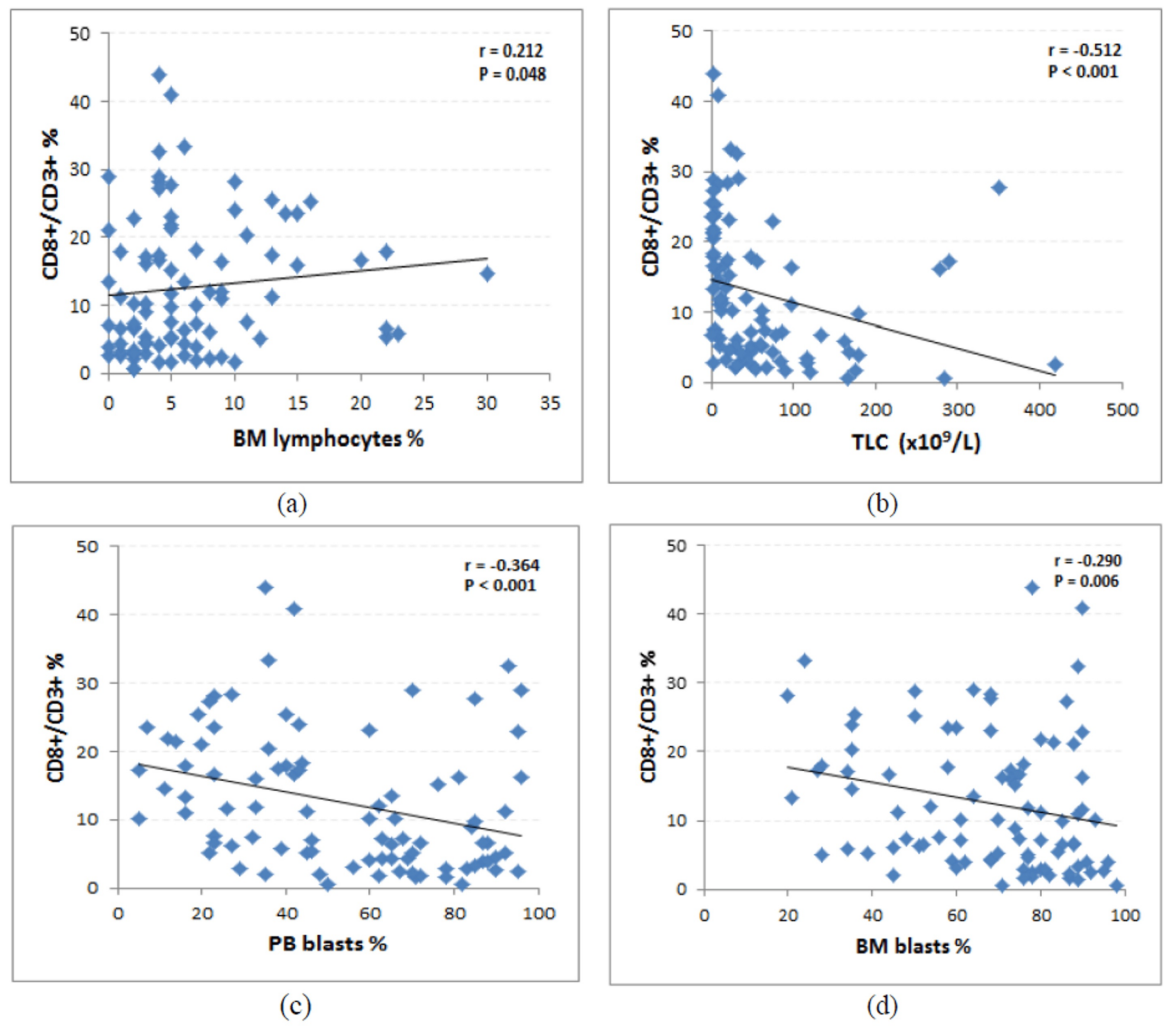
Correlations of BM CD8⁺/CD3⁺ CTL percentage with (a) initial BM lymphocyte percentage, (b) TLC, (c) initial PB blast percentage, and (d) initial BM blast percentage BM, bone marrow; CTL, cytotoxic T-lymphocyte; PB, peripheral blood; TLC, total leukocyte count

BM CD8⁺/CD3⁺ CTL percentage showed a significant positive correlation with initial BM lymphocyte percentage (r = 0.212, p = 0.048) and significant negative correlations with initial TLC (r = -0.512, p < 0.001), initial PB blast percentage (r = -0.364, p < 0.001), and initial BM blast percentage (r = -0.290, p = 0.006) (Figure 2, Table 3).

**Table 3:** Correlations of BM CD8⁺/CD3⁺ CTL percentage with clinical and hematological parameters BM, bone marrow; CTL, cytotoxic T-lymphocyte; Hb, hemoglobin; PB, peripheral blood; PLT, platelets; TLC, total leukocytic count

Parameter		BM CD8⁺/CD3⁺ CTLs (%)
Age	Spearman’s correlation coefficient (r)	0.065
	P value	0.549
PLT (× 10^9^/L)	Spearman’s correlation coefficient (r)	-0.143
	P value	0.184
TLC (× 10^9^/L)	Spearman’s correlation coefficient (r)	-0.512
	P value	<0.001
Hb (g/dl)	Spearman’s correlation coefficient (r)	-0.01
	P value	0.927
PB blasts (%)	Spearman’s correlation coefficient (r)	-0.364
	P value	<0.001
BM blasts (%)	Spearman’s correlation coefficient (r)	-0.29
	P value	0.006
BM lymphocytes (%)	Spearman’s correlation coefficient (r)	0.212
	P value	0.048

Survival analysis

The OS analysis included 84 of 88 cases, as four patients were lost to follow-up. The follow-up period from diagnosis ranged from 0.3 to 20.4 months. The median OS was 1.3 months, with cumulative OS rates of 40.5% at three months, 26.4% at six months, and 21.7% at the end of the study.

Impact of BM CD8⁺/CD3⁺ CTL Percentage on OS

**Figure 3:**
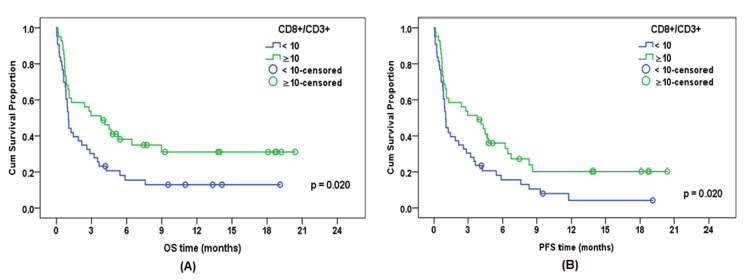
Impact of BM CD8⁺/CD3⁺ T-lymphocyte percentage on survival in 84 AML patients: (A) OS and (B) PFS AML, acute myeloid leukemia; BM, bone marrow; OS, overall survival; PFS, progression-free survival

Patients with BM CD8⁺/CD3⁺ CTLs ≥10% had significantly better OS, with a median of 3.8 months and a cumulative OS of 38.1% at six months, compared with a median of 1.0 month and a cumulative OS of 15.5% at six months in patients with BM CD8⁺/CD3⁺ CTLs <10% (p = 0.020) (Figure 3A).

Impact of BM CD8⁺/CD3⁺ CTL Percentage on PFS

Patients with BM CD8⁺/CD3⁺ CTLs <10% had shorter PFS, with a median of one month and cumulative PFS of 15.5% at six months, compared with a median of 3.8 months and cumulative PFS of 35.9% at six months for patients with BM CD8⁺/CD3⁺ CTLs ≥10% (p = 0.020) (Figure 3B, Table 4).

**Table 4:** Impact of BM CD8⁺/CD3⁺ T-lymphocyte percentage on OS and PFS in 84 AML patients, analyzed using the Kaplan-Meier method with comparisons between groups by the log-rank test AML, acute myeloid leukemia; BM, bone marrow; OS, overall survival; PFS, progression-free survival

Parameter			N	No. of events	Cumulative survival at six months (%)	Median survival (months)	Standard error	95% C	Chi-square (χ²)	P value
OS	CD8⁺/CD3⁺ %	<10	43	37	15.5	1	0.086	0.9-1.2	5.372	0.02
		≥10	41	27	38.1	3.8	1.32	1.2-6.4		
PFS	CD8⁺/CD3⁺ %	<10	43	40	15.5	1	0.086	0.9-1.2	5.376	0.02
		≥10	41	31	35.9	3.8	1.217	1.4-6.2		

## Discussion

Although many studies have focused on the immunophenotypic and genetic aberrations of leukemic cells in AML, which have improved risk stratification, standard therapies have remained largely unchanged over the years. In this context, manipulation of the immune system through various immunotherapeutic approaches, including adoptive T-cell transfer, represents a promising avenue for treatment [8]. Accordingly, we aimed to characterize baseline BM CTLs, a key subset of immune cells in AML, to investigate their clinical significance.

In the present study, no associations were observed between BM CD8⁺/CD3⁺ CTL percentage and patient age, gender, lymphadenopathy, hepatomegaly, or splenomegaly. These findings are consistent with those of El Gendi et al. [11], who reported no significant correlation with age, and Sun et al. [10], who found no association with gender. Similarly, Ismail and Abdulateef [12] reported no significant relationship between CTL subpopulations and organomegaly.

We found that a lower BM CD8⁺/CD3⁺ CTL percentage was associated with adverse prognostic parameters, including TLC ≥ 100 × 10⁹/L (p = 0.011) and hypercellular BM at diagnosis (p = 0.024). BM CD8⁺/CD3⁺ CTL percentage also showed significant negative correlations with initial TLC (r = -0.512, p < 0.001), PB blast percentage (r = -0.364, p < 0.001), and BM blast percentage (r = -0.290, p = 0.006). These findings highlight the potential role of this subset in antitumor immune responses and disease control.

Additionally, BM CD8⁺/CD3⁺ CTL percentage showed a significant positive correlation with initial BM lymphocyte percentage (r = 0.212, p = 0.048). Other studies, however, reported no significant associations between WBC count, platelet count, or BM blast percentage at diagnosis and the CD8⁺ T-cell subset (all p > 0.05) [10,12]. Ismail and Abdulateef [12] also found no significant differences regarding PB blast percentage. In the present study, no association was observed with hemoglobin concentration, which is in agreement with findings by Sun et al. [10] and Ismail and Abdulateef [12].

We further found that a low BM CD8⁺/CD3⁺ CTL percentage was significantly associated with M4/M5 FAB subtypes (p = 0.011). This contrasts with previous studies, which reported no significant differences in CD8⁺ CTL proportions among AML FAB subtypes [11,12].

No associations were detected between BM CD8⁺/CD3⁺ CTL percentage and molecular genetics or cytogenetic risk groups, consistent with prior studies reporting no significant differences regarding prognostic cytogenetic subgroups or FLT3-ITD status (p > 0.05) [12,13]. Similarly, Dange et al. [14] found no significant correlations between CD8⁺ T lymphocytes and cytogenetic or molecular mutation data. Park et al. [6] also reported no significant differences in PB CD8⁺ T lymphocyte percentages among poor, intermediate, and favorable prognostic groups.

In the present study, there was no significant difference in BM CD8⁺/CD3⁺ CTL proportions between responders and non-responders at the end of induction therapy. El Gendi et al. [11] reported similar findings, although they noted that CTLs were higher in responders than non-responders at the end of induction.

Regarding survival outcomes, BM CD8⁺/CD3⁺ CTLs ≥10% were associated with significantly better OS (median 3.8 months; cumulative OS 38.1% at six months) compared with BM CD8⁺/CD3⁺ CTLs <10% (median 1.0 month; cumulative OS 15.5% at six months; p = 0.020). Patients with BM CD8⁺/CD3⁺ CTLs <10% also had shorter PFS (median one month; cumulative PFS 15.5% at six months) compared with those with BM CD8⁺/CD3⁺ CTLs ≥10% (median 3.8 months; cumulative PFS 35.9% at six months; p = 0.020).

These findings differ from previously reported results. Najera Chuc et al. [15] found that increased CD8⁺ T cells were associated with lower one-year OS. Ismail and Abdulateef [12] reported no significant association between CD8⁺ CTLs and relapse, and Alcasid et al. [13] found no significant impact of CD8⁺/CD3⁺ T lymphocyte percentage at diagnosis on OS or disease-free survival. Park et al. [6] also observed no significant influence of PB CD8⁺ T lymphocyte percentages on OS or relapse-free survival, and Dange et al. [14] reported no significant differences in CD8⁺ T lymphocyte percentages between patients who achieved remission, died during induction, or had refractory disease.

Overall, our results suggest that a higher BM CD8⁺/CD3⁺ CTL percentage at diagnosis is significantly associated with improved survival outcomes in AML patients, highlighting the potential prognostic relevance of this immune cell subset.

## Conclusions

Our results suggest that the BM CD8⁺ CTL subset in the studied AML cases represents a crucial component of the immune cell composition, showing significant associations with certain prognostic hematological and clinical parameters, as well as response to therapy. Discrepancies between our findings on disease outcomes and those reported in the literature may, in part, reflect differences in treatment protocols and follow-up periods. The relatively small number of patients in some subgroups and the short follow-up duration are major limitations of this study. Small subgroup sizes may limit the statistical power of analyses, while shorter follow-up periods may underestimate long-term risks and obscure treatment effects. Therefore, further studies are warranted to expand upon our current findings and to explore potential therapeutic applications. Investigations that include functional analyses are particularly needed to better define the role of BM CD8⁺ CTLs in AML pathogenesis and prognosis.
